# Nanoparticle-based biomolecules in cancer diagnosis, therapy, drug delivery and prognosis

**DOI:** 10.3389/fdmed.2024.1482166

**Published:** 2024-11-21

**Authors:** Sowmya SV, Dominic Augustine, Jagdish Hosmani, Francesco Pagnoni, Rodolfo Reda, Luca Testarelli, Shankargouda Patil

**Affiliations:** ^1^ Department of Oral & Maxillofacial Pathology and Oral Microbiology, Faculty of Dental Sciences, MS Ramaiah University of Applied Sciences, MSR Nagar, Bengaluru, India; ^2^ Department of Diagnostic Dental Sciences, Oral Pathology Section, College of Dentistry, King Khalid University, Abha, Saudi Arabia; ^3^ Department of Oral and Maxillofacial Sciences, Sapienza University, University of Rome, Rome, Italy; ^4^ College of Dental Medicine, Roseman University of Health Sciences, South Jordan, UT, United States; ^5^ College of Graduate Studies, Roseman University of Health Science, South Jordan, UT, United States

**Keywords:** cancer diagnosis, cancer therapy, liposomal vaccines, nanochemodrugs, nanoparticles, nano-pharmaceuticals, prognosis

## Abstract

**Introduction:**

Nanoparticles have orchestrated a paradigm shift in the landscape of cancer diagnosis and therapy, presenting a multifaceted approach to tackle the intricacies of malignancies. This comprehensive exposition delves deep into the forefront of nanomedicine, elucidating pivotal strategies and innovations primed to metamorphose the domain of cancer management.

**Methodology:**

Nanoparticles transcend traditional boundaries, enabling meticulous, site-specific drug release while minimizing systemic toxicity. Intricately designed activation mechanisms, encompassing pH and enzymatic responsivity, along with concentration-dependent strategies, exploit the distinctive attributes of cancer cells, heralding an era characterized by unprecedented therapeutic precision. The pervasive influence of nanotechnology extends to diagnostics, unlocking the realm of early disease detection and personalized treatment. These versatile agents bestow empowering capabilities upon sensitive imaging modalities, affording real-time monitoring and theranostic potential.

**Results:**

This exposition showcases the evolution of cutting-edge nanoplatforms, bridging the chasm between diagnosis and therapy, thereby redefining the confines of cancer care. This review elucidates strategies to combat drug resistance, a perennial challenge within cancer management. By targeting efflux transporters, modulating apoptotic pathways, and countering hypoxia-induced resistance, nanoparticles stand at the vanguard of therapeutic innovation, poised to reinvigorate treatment efficacy.

**Discussion & Conclusion:**

Moreover, this exposé underscores the imminent clinical translation of nanoparticle-based drugs, accentuating their potential to metamorphose the landscape of cancer management. Liposomal vaccines, nano-pharmaceuticals, and nanochemodrugs, currently navigating the crucible of clinical trials, bear immense promise in advancing the realm of precision medicine. In this epoch of precision medicine, nanoparticle-fueled innovations stand poised to propel cancer diagnosis and therapy to unprecedented peaks.

## 1 Introduction

In the ever-evolving landscape of contemporary oncology, there is a profound synergy, uniting cutting-edge nanotechnology with the intricate world of biomolecular science. This dynamic partnership has paved the way for unprecedented progress in cancer diagnosis, therapy, drug delivery, and prognosis (1). Nanotechnology, wielding dominion over structures and materials at the nanoscale, has revolutionized our perception and approach to the challenges posed by cancer. Nanoparticles are diminutive structures with particle sizes ranging from 1 to 1,000 nm with unique physicochemical properties that can defy conventional boundaries and improve the efficacy of cancer therapeutics (2).

According to recent statistics, cancer is the second leading cause of death globally, with an estimated 10 million deaths and approximately 19.3 million prognoses in the year 2020. The multifaceted nature of cancer is characterized by genetic diversity, intercellular heterogeneity, and dynamic microenvironments, necessitates innovative approaches. While conventional therapeutic modalities, to varying extents, prove effective, they often lack the precision and efficacy demanded by this formidable malignancy. The nanoparticle-based biomolecules, meticulously designed for exquisite specificity and tailored to navigate the intricate labyrinth of cancer biology have demonstrated improved pharmacokinetics, precise targeting, and ability to overcome drug resistance (3).

Early detection of cancer plays a pivotal role in its successful management. Conventional diagnostic methods, however, harbor limitations, frequently detecting malignancies at advanced stages when therapeutic options are scant. Herein, the integration of nanoparticles as diagnostic probes sparked a revolution. These nanoparticles conjugated with targeting ligands and contrast agents, empower the detection of minuscule cancerous lesions, even at the molecular level. Recent advancements have witnessed the development of sophisticated nanoparticle-based biosensors and imaging modalities, elevating not only the sensitivity and specificity of cancer detection but also equipping clinicians with real-time information to guide treatment decisions (3).

The conventional challenges associated with drug delivery in cancer treatment have been ingeniously transformed into opportunities through nanoparticle-based carriers. These nanoscale vehicles, armed with the capacity to encapsulate and transport a plethora of therapeutic agents, navigate the complex tumor microenvironment with surgical precision. They capitalize on the enhanced permeability and retention effect, facilitating the preferential accumulation of drugs within tumors (1). Furthermore, advances in stimuli-responsive nanoparticles and targeted drug delivery systems have opened new vistas for therapeutic customization and optimization. This review will elucidate recent breakthroughs in nanoparticle-based therapies, including the advent of immunomodulatory nanoparticles that harness the body's immune system to combat cancer.

The convergence of nanotechnology and biomolecular science, exemplified by nanoparticle-based biomolecules, stands as a beacon of hope in the relentless battle against cancer. As the boundaries of nanoparticle-based cancer therapeutics continue to expand, collating, synthesizing, and critically evaluating these advancements becomes essential. This review will navigate through the recent findings and advancements, illuminating the path toward a future where the intricate tapestry of cancer is unraveled, and tailored, and precision therapies become the cornerstone of oncology.

## 2 Search methodology

Databases such as Scopus, PubMed and Web of Science was searched using the key words “(Nanoparticles) AND (cancer diagnosis)) AND (Therapy and Prognosis)) AND (Drug Delivery)” The review was conducted by 2 reviewers (SVS and DA). Inclusion criteria included articles that focused on nanoparticle-based biomolecules, articles included in the review contained text regarding nanoparticles in cancer diagnosis, therapy, drug delivery and prognosis. Those articles that concerned nanoparticles but however did not focus on their role in cancer diagnosis, therapy, drug delivery and prognosis were excluded. A total of 30 articles were shortlisted to extract and analyse the content from literature published in the last 10 years.

## 3 Nanoparticles for Cancer Diagnosis and Screening

**Figure 1:**
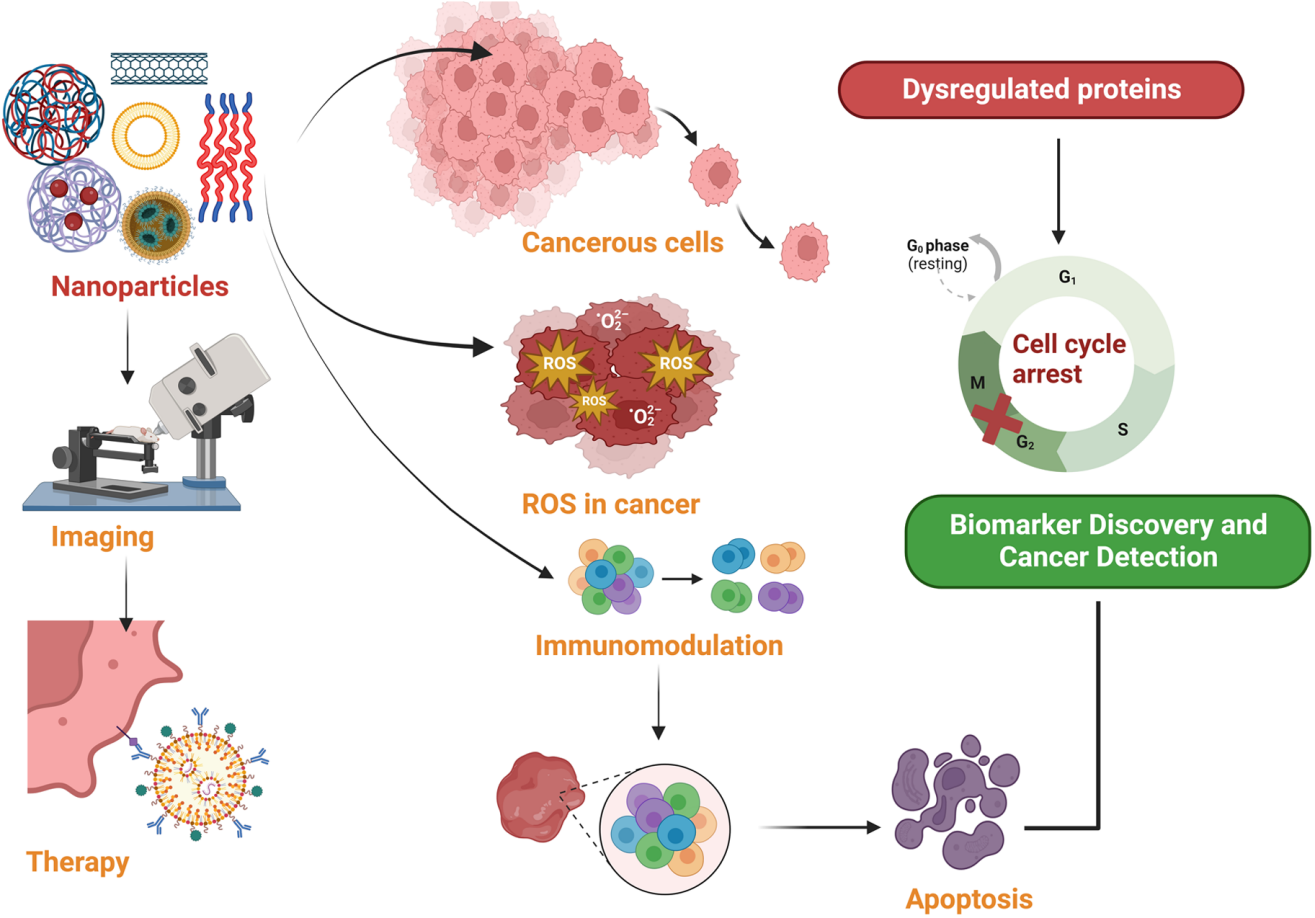
The therapeutic potential of nanoparticles in cancer management.

Nanoparticles, particularly Nanotheranostics, which employs complex nanoparticles made from materials such as gold, iron oxide, silica, etc. have emerged as a promising tool for cancer diagnosis and treatment (Table 1). Nanoparticles have emerged as promising tools in the realm of cancer diagnostics, offering the capability to capture cancer biomarkers like cancer-associated proteins, circulating tumor DNA (ctDNA), circulating tumor cells, microRNA, and extracellular vesicles with exceptional precision and efficiency. This innovative approach capitalizes on the unique characteristics of nanoparticles, particularly their remarkably high surface area-to-volume ratio when compared to conventional bulk materials (2). This inherent property of nanoparticles is instrumental in enhancing their effectiveness for cancer diagnosis (Figure 1). However, despite their immense potential, the utilization of biomarkers in cancer diagnosis faces several formidable challenges. One such challenge is the often-low concentrations of these biomarkers in bodily fluids, making their detection and quantification a demanding task. Furthermore, there exists considerable variability in the abundance and timing of biomarkers among individual patients, complicating the diagnostic process (1).

**Table 1:** Nanoparticle-based biomarker discovery.

Category	Nanoparticle types used	Outcome	Cancer cells targeted
Nanoparticles for biomarker discovery (4–8)			
Protein biomarkers	Gold nanoparticles, quantum dots, silica nanoparticles, iron oxide nanoparticles	Detection of specific cancer-related proteins such as PSA, HER2, CA-125, EGFR, AFP, CA-19-9, PSMA, HER3, BRCA, etc.	Various cancer types including breast, prostate, ovarian, lung, and pancreatic cancer.
DNA methylation detection	Quantum dots, magnetic nanoparticles, carbon nanotubes, silica nanoparticles	Identification of DNA methylation patterns associated with cancer.	Detection of epigenetic alterations in various cancers, including colorectal and breast cancer.
ctDNA detection	Magnetic nanoparticles, gold nanoparticles, polymer nanoparticles	Isolation and detection of circulating tumor DNA (ctDNA) from blood samples.	Effective in detecting ctDNA from patients with lung, breast, colorectal, and other cancers.
microRNA detection	Lipid-based nanoparticles, silica nanoparticles, polymer nanoparticles	Profiling and detection of cancer-related microRNA signatures (miR-21, miR-155, miR-34a, miR-155-5p, miR-10b, miR-221/222, miR-125b, etc.)	Identification of microRNA biomarkers in various cancers, including lung, ovarian, and prostate cancer.
Extracellular vesicle detection	Exosome-mimicking nanoparticles, polymer nanoparticles, liposomes	Capture and analysis of extracellular vesicles for non-invasive cancer diagnosis (Exosomes, microvesicles, apoptotic bodies, once-exosomes, etc.)	Detection of extracellular vesicles secreted by cancer cells in multiple cancer types.
Nanoparticles for detection of cancerous cells (4–8)			
Detection of circulating tumour cells	Magnetic nanoparticles, gold nanoparticles, polymer nanoparticles	Isolation and identification of circulating tumour cells (CTCs) in blood samples.	Effective in capturing CTCs from patients with breast, prostate, colorectal, and other cancers.
Detection through cell surface proteins	Quantum dots, gold nanoparticles, magnetic nanoparticles	Identification of specific cell surface protein markers on cancer cells (EpCAM, HER2, EGFR, CD20, CD19, PD-L1, CA-125, CD-33, CD-44, etc.)	Targeting cell surface proteins associated with breast, ovarian, prostate, and other cancer types.
Detection based on mRNA	Carbon nanotubes, silica nanoparticles, polymer nanoparticles	Selective capture and detection of cancer-related mRNA transcripts (FOXM1, c-KIT, BCL-2, c-MYC, ERCC1, etc.)	Detection of mRNA markers in various cancer cells, including lung, pancreatic, and liver cancer.

### 3.1 Role of nanoparticles in biomarker discovery

In the domain of protein detection, nanoparticles like Quantum dots (QDs), gold nanoparticles (AuNPs), and polymer dots (PDs) have emerged as invaluable assets. These nanoparticles find application in sandwich-type assays, enabling the detection of proteins by interacting with antibodies or aptamers. Among these, Quantum dot (QD) based biosensors stand out, owing to their distinctive optical characteristics, as they excel in detecting protein biomarkers such as neuron-specific enolase (NSE) and carcinoembryonic antigen (CEA). Moreover, zinc oxide (ZnO) QD-based immunoassays exhibit remarkable promise in monitoring cancer treatment progress and predicting tumor recurrence. The synergy of QDs with various techniques, including staining and fluorescence, has substantially elevated the precision and sensitivity of protein biomarker detection. Active targeting of cancerous tissues *in vivo* is achieved through the use of peptides, such as the Arg-Gly-Asp (RGD) motif, facilitating precise tumor localization. Aptamers, which are single-stranded DNA or RNA sequences, contribute their high affinity and specificity to the arsenal of cancer detection tools. When conjugated with nanoparticles, aptamers form a potent platform for recognizing cancer cells, exemplified by the A10 RNA aptamer-conjugated polymeric nanoparticles. Furthermore, upconverting nanophosphors (UCNPs) have emerged as promising luminous labels in biological contexts, significantly enhancing the detection sensitivity of cancer-related biomolecules (4).

### 3.2 Role of nanoparticles in DNA methylation and ctDNA detection

Circulating tumor DNA (ctDNA), which comprises fragments of tumor-derived DNA circulating in the bloodstream, has revolutionized early cancer detection by enabling the identification of cancer-specific genetic anomalies. In this groundbreaking field, nanoparticles have emerged as instrumental allies in the highly sensitive detection of ctDNA, achieved through their interaction with nucleic acid probes. One notable example is the utilization of DNA silver nanoclusters, a powerful tool for detecting specific genetic mutations associated with breast cancer. This approach has demonstrated remarkable sensitivity and selectivity, marking a significant stride in the early diagnosis of this devastating disease (5).

### 3.3 Role of nanoparticles in microRNA profiling and extracellular vesicles analysis

The dynamic realm of microRNA (miRNA) detection has harnessed the capabilities of nanoparticles to dissect and unveil cancer-associated miRNA signatures. Notably, nanoparticles such as semiconducting polymer dots (PDs) have exhibited their prowess in effectively and precisely labeling cellular targets, thereby enabling the discernment of miRNA biomarkers. Nanotechnology has brought about a profound transformation in the realm of detecting DNA methylation patterns linked to cancer. It has ushered in rapid, discerning, and highly sensitive electrochemical or colorimetric assays, revolutionizing the identification of methylation patterns as prevalent cancer biomarkers. Extracellular vehicles (EVs), reservoirs of diverse biomolecules, provide invaluable insights into the intricacies of cancer biology. The application of nanotechnology has streamlined the process of isolating and analyzing EVs, furnishing a non-invasive avenue for detecting cancer-related vesicles released by malignant cells. Innovations like magnetic nanopore capture and advanced sensor platforms have enabled the isolation and comprehensive profiling of EVs, thereby facilitating the identification of miRNA biomarkers and advancing our comprehension of tumor cell heterogeneity (6).

### 3.4 Role of nanoparticles in the detection of tumour cells

Nanotechnology has made a profound impact on the realm of cancer cell detection, with a particular focus on circulating tumor cells (CTCs) that bear critical significance in early cancer diagnosis and prognosis. The deployment of nanoparticles, including magnetic nanoparticles (MNPs), gold nanoparticles (AuNPs), quantum dots (QDs), and various nanostructures, has ushered in enhanced methods for capturing and identifying CTCs. Immunomagnetic nanoparticles, cleverly functionalized with antibodies, exhibit a remarkable ability to specifically target CTCs expressing surface markers like EpCAM, thus marking a significant advancement in this field. In addition to targeting surface markers, nanoparticles have revolutionized cancer cell detection through the recognition of cell surface proteins. The spotlight has been on EpCAM, which has been successfully targeted using anti-EpCAM molecules, enabling the efficient screening of CTCs. Researchers have also explored alternative markers, such as vimentin, androgen receptor, and glycan, to overcome the limitations of EpCAM-based detection. These markers offer improved specificity, catering to different cancer types and stages, including mesenchymal CTCs often associated with metastatic cancer (7).

The detection of cancer cells has taken a further leap with the integration of nanotechnology in mRNA marker-based approaches. Nanoflares, characterized by their gold nanoparticle probes modified with oligonucleotides, have been instrumental in achieving sensitive detection of intracellular mRNA. Nanoflares empower researchers to quantify mRNA transcripts within living cells with exceptional sensitivity and signal-to-noise ratios, thereby facilitating the profiling of genetic information at the single-cell level. The development of plasmonic nanoparticle networks has provided a promising avenue for detecting single mRNA variants. This cutting-edge technique empowers the quantification and differentiation of mRNA splice variants with single-copy sensitivity, opening new horizons for the future of single-cell genetic profiling (8).

## 4 Nanomaterials for cancer therapy

**Figure 2:**
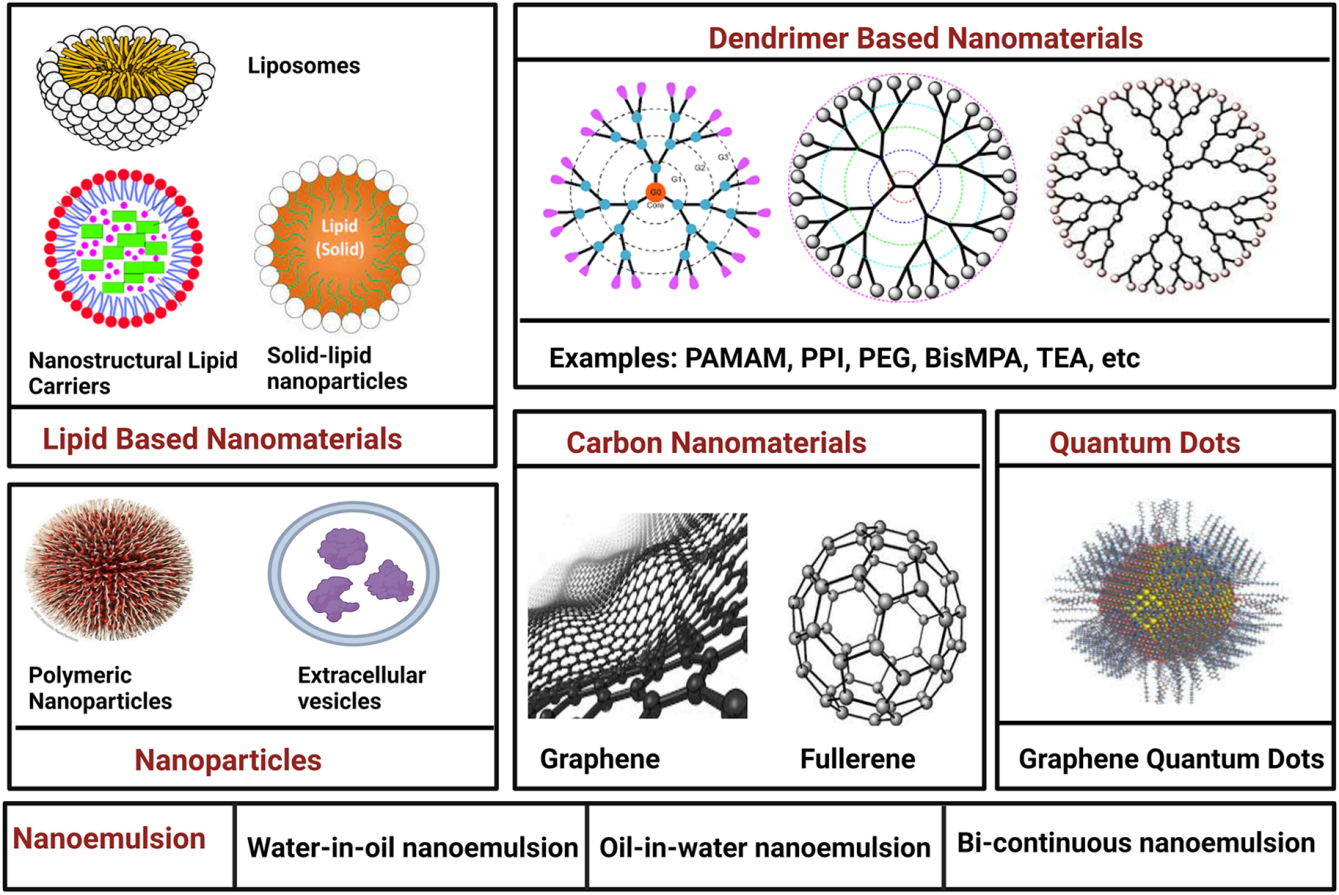
Nanomaterials utilized in cancer therapy.

Medical nanotechnology, operating at the nanoscale (1–100 nm), is instrumental in designing therapeutic drugs and devices with unique optical, magnetic, and electrical properties. Nanomaterials, characterized by high surface-to-volume ratios, enhanced conductivity, and distinctive features, find applications in drug delivery, controlled release, and improved biocompatibility for crossing biological barriers (9). Nanomaterials hold immense promise in cancer therapy, offering precise drug delivery while minimizing harm to healthy cells. Emerging therapies like photodynamic therapy (PDT) and photothermal therapy (PTT) show potential. PDT employs cancer-specific photosensitizers activated by light to induce cancer cell death. PTT uses nanomaterials to raise the temperature in cancerous areas, killing cancer cells. Superparamagnetic nanomaterials like superparamagnetic iron oxide nanoparticles (SPION) hold promise for cancer diagnosis and treatment, particularly hyperthermia therapy. Nanomaterial-based cancer therapy enhances drug delivery, improving drug stability, solubility, and targeting (10). Passive targeting capitalizes on the enhanced permeability and retention (EPR) effect, while active targeting involves conjugating nanomaterials with antibodies, peptides, aptamers, or small molecules (Table 2). Despite limited clinical adoption, ongoing research in cancer pathology and nanoscience continues to advance effective cancer treatments and diagnostics. The nanomaterials utilized in cancer therapy are discussed in the following section (Figure 2).

**Table 2:** Nanomaterials for the improved anticancer drug delivery (9–19).

Nanomaterials	Payload	Therapies involved	Targeted cancer model	Outcome
PLGA-NP	PTX	Chemotherapy	Prostate cancer PC3	Improved drug delivery efficiency compared with free PTX.
	Alantolactone erlotinib	Targeted therapy	Pancreatic cancer	Induction of significant cancer cell apoptosis and anticancer effect.
PEG, transferrin modified NP	Nucleic acids	Nucleic acid therapy	Leukemia	Improved transfection efficiency in K562 leukemia cells.
Tmab modified NP	Docetaxel	Chemo and targeted therapy	HER2 +/- cells	Increased cytotoxicity in HER2-positive BT474 cells but not in HER2-negative MCF7 cells.
	Paclitaxel		Breast cancer (HER2- cell lines)	Improved treatment efficacy and reduced cytotoxicity to human breast epithelial cell control.
Exosomes	Doxorubicin	Chemotherapy	Breast cancer Ovarian cancer	Enhanced doxorubicin cytotoxicity and reduced drug accumulation in mouse heart.
AuNP-encapsulated IONPs/Ag cores	IONPs/Ag	Photothermal therapy	Melanoma	Effective MRI T2 contrast agent and efficient photothermal therapy agent.
Liposome	Gemcitabine	Chemotherapy	Human pancreatic cancer cell line	Enhanced drug delivery and inhibition of tumor growth.
SLN	Paclitaxel	Chemotherapy	Ovarian cancer	Improved drug delivery and increased cytotoxicity against cancer cells.
NLC	Doxorubicin	Chemotherapy	Breast cancer	Enhanced drug encapsulation, sustained release, and increased cytotoxicity.
Nano-emulsion	Docetaxel	Chemotherapy	Non-small cell lung cancer	Improved drug solubility, intracellular uptake, and enhanced cytotoxicity.
PAMAM dendrimers	Methotrexate	Targeted therapy	Breast cancer	Selective drug delivery and inhibition of cancer cell growth.
PPI dendrimers	Doxorubicin	Chemotherapy	Breast cancer	Improved drug solubility, intracellular uptake, and enhanced cytotoxicity.
PEG dendrimers	Breast cancer	Targeted therapy, chemotherapy	Ovarian cancer	Enhanced drug delivery to cancer cells and improved cytotoxicity.
Graphene	Doxorubicin	Chemotherapy	Breast cancer	Improved drug delivery and enhanced cytotoxicity.
Fullerenes	C60-based compounds	Various	*In-vitro* studies	Potential application in drug delivery.
Cylindrical nanotubes	Paclitaxel	Chemotherapy	Lung cancer	Improved drug delivery.
Graphene Quantum dots	Curcumin	Targeted therapy	Colon cancer	Improved drug delivery and enhanced cytotoxicity.
Nanodiamonds	Cisplatin	Chemotherapy	Ovarian cancer	Improved drug delivery.
Metallic nanomaterials	Gold Nanoparticles	Photothermal Therapy (PTT)	Breast cancer	Effective photothermal therapy and potential for imaging.
Magnetic nanomaterials	iron oxide nanoparticles	magnetic hyperthermia	Glioblastoma	Induced hyperthermia for cancer cell ablation.

### 4.1 Nanoparticles

Polymeric nanoparticles (PNPs), alongside other nanoparticle types, have garnered significant attention within the field of cancer therapy. These colloidal macromolecules, with sizes spanning 10–1,000 nm, excel as drug carriers, facilitating controlled drug release at precise cancer sites. Initially, nonbiodegradable polymers like polymethyl methacrylate and polystyrene found use in PNP fabrication, but concerns regarding toxicity and chronic inflammation prompted the development of biodegradable alternatives such as polylactic acid (PLA), poly (lactic-co-glycolic acid) (PLGA), and natural polymers like chitosan and gelatin. These biodegradable PNPs offer enhanced stability, and diverse administration methods, and safeguard drugs against degradation, thus minimizing toxicity to normal tissues. Notably, PNPs loaded with cisplatin have emerged as a solution in chemotherapy, effectively mitigating cisplatin-induced cytotoxicity (11). In drug delivery, two principal methods exist: passive targeting and active targeting. Passive targeting leverages the enhanced permeability and retention (EPR) effect, enabling chemical drugs to infiltrate tumor sites through leaky blood vessels. Conversely, active targeting entails attaching targeting polymers to PNPs, thereby elevating bioavailability. Surface coatings, such as polysorbates, have demonstrated their ability to enhance bioavailability by facilitating interactions with blood-brain barrier endothelial cell membranes (1).

Monoclonal antibodies (mAbs) have also wielded substantial influence in cancer therapy. By conjugating mAbs with cytotoxic drugs, a strategy known as antibody-drug conjugates (ADCs), it becomes possible to achieve heightened specificity and reduced toxicity. An illustrative example involves trastuzumab (Herceptin), employed in ADC systems to treat HER2-positive breast cancer, delivering improved therapeutic efficacy in comparison to trastuzumab used in isolation (11). Extracellular vehicles (EVs), including exosomes, have emerged as natural carriers for diverse anti-tumor compositions. Spanning sizes from 50 to 1,000 nm, these vesicles encompass proteins, RNA, and DNA, rendering them ideal for gene therapy applications. Exosomes possess the capability to transport nucleic acids, small molecules, and proteins, thereby enhancing cytotoxicity, preventing drug accumulation in the heart, and inhibiting tumor growth. Nevertheless, challenges persist, including the lack of standardized criteria for exosome isolation, obscure mechanisms underlying cancer treatment, and the intricacies of exosome preservation (2).

### 4.2 Lipid-based nanomaterials

Lipid-based nanomaterials have witnessed remarkable progress, with three primary categories on the forefront: liposomes, solid lipid nanoparticles (SLNs), and nanostructured lipid carriers (NLCs). The unique structure of liposomes comprises a hydrophilic core and a hydrophobic phospholipid bilayer, enabling them to encapsulate both hydrophilic and hydrophobic drugs. This characteristic safeguards drugs from degradation during circulation in the bloodstream. Liposomes come in various forms, including unilamellar and multilamellar vesicles. Over time, researchers have addressed pivotal challenges in liposome development, such as traversing biological barriers and evading rapid clearance by the mononuclear phagocyte system (MPS). Strategies like membrane modification, involving proteins, peptides, or polymers, have extended liposome half-lives. Stealth liposomes, featuring polyethylene glycol (PEG) conjugation, have displayed prolonged longevity, rendering them suitable for applications such as Kaposi's sarcoma treatment. Efficient drug loading and controlled release stand as crucial aspects of liposome nanocarrier design. Particularly in the context of cancer chemotherapy, enhancing bioavailability is vital for heightened drug efficacy. Liposomes can be finely tuned for co-delivery and controlled release of various agents, including small-molecule inhibitors and siRNA molecules, showcasing synergistic effects and improved therapeutic outcomes (12).

Solid lipid nanoparticles (SLNs) chart another path in nanocarrier development. Distinguished as “*zero-dimensional”* nanomaterials, SLNs differ from liposomes by incorporating solid lipid components. This choice enhances stability and prolongs drug release. However, challenges such as gelation and low incorporation rates may arise due to their crystalline structure. Nanostructured lipid carriers (NLCs), a more recent innovation, amalgamate the strengths of both liposomes and SLNs. These carriers consist of a core matrix loaded with solid and liquid lipids, offering superior stability, loading capacity, biocompatibility, and drug protection. NLCs prove versatile through various administration methods, making them particularly valuable for lipophilic drug compounds employed in cancer treatment (12, 13).

### 4.3 Nano-emulsions

Nanoemulsions (NEs) stand as a prominent category within colloidal nanoparticles, comprising an aqueous phase, emulsifying agents, and oil. These minute spheres possess an amorphous, lipophilic surface adorned with a negative charge, offering distinct advantages over other lipid-based nanomaterials and nanoparticles. The benefits of NEs encompass optical clarity, thermodynamic stability, a generous surface area, facile manufacturing, biodegradability, and an optimal drug release profile. A compelling avenue of research involves tailored NEs with membranes, unlocking promising possibilities. Co-delivery strategies employing NEs enhance drug bioavailability and efficacy. For example, NEs loaded with spirulina polysaccharides and paclitaxel (PTX) hold the potential to fortify PTX's anti-tumor impact by modulating immunity pathways. NEs also hold promise in immunotherapy, where they can house immune-stimulating agents like Interferon-gamma (IFN-γ). These modified NEs exhibit stability and the capacity to incite cellular responses against cancer cells, showcasing their applicability in cancer treatment (14). NEs offer a strategy to combat multidrug resistance (MDR) encountered in some cancer cells, often due to ATP-binding cassette transporters (ABCs) that expel anticancer drugs. NEs, co-delivering multiple drugs such as baicalein and paclitaxel, present a potential solution by elevating oxidative stress and heightening cell sensitivity to paclitaxel, potentially overcoming MDR. However, translating NEs into clinical applications presents challenges. The production process often demands high-temperature and high-pressure conditions, limiting the choice of starting materials. The energy-intensive nature of NE preparation renders it costlier than traditional formulations. Additionally, a deeper comprehension of NE chemistry, component interactions, and *in vivo* metabolism is crucial to ensure their safety and efficacy in clinical contexts. Despite these hurdles, NEs hold exciting prospects in drug delivery and cancer therapy (15).

### 4.4 Dendrimers

Dendrimers, distinctive macromolecules with highly branched surfaces typically ranging from 1 to 10 nm, have garnered attention in cancer therapy. Comprising central cores for drug loading, interior dendritic structures, and functionalized exteriors, dendrimers like PAMAM, PPI, PEG, Bis-MPA, 5-ALA, and TEA offer unique advantages. Their precise structure yields defined molecular weight, adjustable branches, low polydispersity, and hydrophobic drug solubility. Cationic dendrimers effectively form nucleic acid complexes, making them potent carriers. In a nanohybrid system, combining PAMAM dendrimers and carbon dots, multidrug resistance (MDR) management and cancer cell monitoring via fluorescence imaging were achieved. Complexes between blue-emitting carbon dots and the anticancer drug DOX, along with dendrimers functionalized with targeting ligands and drug efflux inhibitors, showed promising anti-cancer effects. Dendrimers excel in combination therapies, co-delivering DOX and TRAIL plasmids for enhanced antitumor efficacy. PAMAN dendrimers explored for combined chemotherapy and photothermal liver cancer cell treatment hold potential despite limitations like low transfection efficiency and cellular internalization (16).

### 4.5 Carbon nanomaterials

Carbon nanomaterials (CNMs) represent a diverse group of nanoscale carbon-based materials celebrated for their unique electronic, thermal, optical, and mechanical properties. In the context of cancer theragnostics, CNMs offer a safer and more biocompatible alternative to metal-based nanomaterials. Their innate hydrophobicity equips them as effective drug delivery platforms, capable of loading chemical drugs through mechanisms like π–π stacking and hydrophobic bonding. A range of CNMs have undergone intensive scrutiny for their potential in cancer treatment, including graphenes, fullerenes, carbon nanotubes (CNTs), carbon nanohorns (CNHs), carbon quantum dots (CQDs), and graphyne (GDY). Each CNM boasts unique morphological structures and properties. For instance, graphene, a two-dimensional crystal with remarkable mechanical and electronic attributes, finds applications in biomedicine. Different types of graphene-based nanomaterials, such as single-layer graphene, multi-layer graphene, graphene oxide (GO), and reduced graphene oxide (rGO), offer distinct advantages for cancer theragnostic (17).

GO, a chemically modified derivative of graphene stands out in cancer therapy due to its enhanced biocompatibility and stability. Functional oxygen groups enhance their hydrophilicity, prevent aggregation in aqueous solutions, and facilitate efficient drug loading, enabling various cancer treatment strategies like targeted therapy, photodynamic therapy (PDT), photothermal therapy (PTT), and cancer diagnosis (17). Fullerenes, including C60 and C70 molecules, possess properties like free radical scavenging and unique attributes for PDT and PTT, making them promising candidates for cancer therapy. CNTs, cylindrical tubes formed from rolled graphene, hold promise in strategies enhancing the immune response in cancer treatment due to their immunostimulatory potential. They also serve as efficient platforms for PDT and PTT. CNHs, belonging to the carbon allotrope family, share similarities with CNTs. With surface modifications to enhance bioavailability, they offer drug-loading and photothermal capabilities, contributing to the development of drug-delivery systems with combined features. However, concerns about CNM toxicity and side effects in cancer therapy persist. Factors like surface modification, concentration, size, and shape influence CNM toxicity. Further research is imperative to unravel the mechanisms and central aspects of cellular toxicity associated with CNMs (17, 18).

### 4.6 Quantum dots

Quantum dots (QDs) have emerged as versatile biomedical imaging agents, capitalizing on their unique optical and electronic properties. These nanometer-scale semiconductor crystals have revolutionized biological fluorescence imaging, outperforming traditional organic fluorophores. QDs offer tunable fluorescence emissions spanning visible to infrared wavelengths, high absorption coefficients, and exceptional photostability. The carbon-based QDs, including graphene quantum dots (GQDs), nanodiamonds, and carbon dots (CDs), their applications in cancer imaging and sensing are particularly promising. GQDs, in particular, have gained prominence due to their extensive surface area, biocompatibility, and rapid excretion. They have been pivotal in developing photoluminescent glycodendrimers for targeted drug delivery, enhancing cancer cell destruction, and serving as photoluminescent imaging agents. GQDs have also been used for targeted therapy by conjugating them with folic acid, facilitating uptake into folate receptor (FR)-positive cancer cells. Beyond bioimaging and biosensing, modified GQDs have shown potential in photothermal therapy (PTT) and photodynamic therapy (PDT), effectively ablating tumor cells under NIR-II irradiation. Additionally, carbon quantum dots (CDs) and nanodiamonds contribute to targeted therapy, PDT, cancer imaging, and modulation of antitumor immunity (19).

## 5 Nanoparticle activation methods in cancer treatment

Precise cancer treatment localization is key to mitigating the often-severe side effects of cancer therapies. It enables the administration of higher, potentially harmful doses directly to the tumor site, sparing healthy tissues. Achieving controlled release or activation of therapeutic compounds or nanoparticles necessitates the development of intelligent systems. These systems can be modulated based on distinctions between cancerous and healthy cells or triggered externally by the cell's microenvironment (20).

### 5.1 Intrinsic activation

Cancer cells have higher intracellular pH (around 7.3–7.6) and lower extracellular pH (around 6.8–7.0) compared to healthy cells (7.2 and 7.4, respectively). This acidic tumor environment activates enzymes and influences gene expression, resulting in distinct differences in protein and enzyme levels (20).

#### 5.1.1 Nanoparticle activation through altered pH

pH-activated nanoparticles represent a groundbreaking approach that capitalizes on pH variations within the body to deliver targeted therapies, minimizing harm to healthy cells and maximizing therapeutic impact. Different organs, such as the stomach and intestines, have unique pH levels, posing challenges for drug delivery. pH-sensitive systems like solid lipid nanoparticles and polymeric materials offer solutions by releasing drugs in less acidic environments, ensuring precise drug concentrations where needed. Tumor tissues, known for their lower pH, enable smart nano-systems to release cargo specifically within malignant environments. These systems employ pH-responsive materials to release drugs upon encountering the acidic microenvironment of tumors, improving targeting accuracy and minimizing side effects. Cancer cells themselves exhibit distinct pH characteristics compared to healthy cells, particularly within lysosomal compartments. pH-sensitive nanomaterials, including polymers, metals, and lipids, are designed to release therapeutic compounds selectively within cancer cells while sparing healthy ones. These innovative pH-activated nanoparticles not only deliver chemotherapeutics but also nucleic acids for gene therapy, expanding their therapeutic potential. With diverse applications and proven efficacy in preclinical studies, from iron oxide nanotubes to calcium-based materials, pH-activated nanoparticles lead the way in cancer treatment innovation, offering enhanced therapeutic outcomes and reduced side effects (Table 3) (21).

**Table 3:** pH-activated nanoparticles in cancer treatment (20, 21).

Nanoparticles	Targeted release mechanism	Specific application	Release location	Drug	Efficacy
Solid lipid nanoparticles	pH-responsive release system in the intestine	Oral delivery of chemotherapeutic agents (doxorubicin)	Intestine (higher pH)	Doxorubicin (DOX)	High drug loading (785.7 mg DOX per 1 g of SLN), cytotoxicity observed (over 30% after 24 h)
Self-microemulsifying drug delivery system	pH-responsive release in the gastrointestinal tract	Oral delivery of natural products (curcumin)	Gastrointestinal tract (pH-responsive)	Curcumin	Increased water solubility, higher adsorption rate (over 90% within 12 h)
pH-activated nanovalves	Lysosomal acidic pH triggers valve activation	Intracellular release of anti-cancer drugs (e.g., doxorubicin)	Lysosomes (pH-responsive)	Doxorubicin (DOX)	High cellular toxicity (over 80% cell death in cancer cells)
pH-responsive metallic nanomaterials (e.g., titanium perioxide, iron oxide)	pH-responsive metallic nanomaterials (e.g., titanium perioxide, iron oxide)	pH-responsive drug release at tumor microenvironment	Lung cancer models (TiOx/DOX), Carcinoma cells (iron oxide nanotubes)	Tumor microenvironment (pH-responsive)	Chemotherapeutic drugs (e.g., DOX, Paclitaxel)
Calcium-based nanoparticles	Calcium-based nanoparticles	Rapid degradation in acidic environments	Delivery of photosensitizers and chemotherapeutics {Mn2+-chelated chlorin e6 [Ce6(Mn)], DOX}	Acidic environment (pH = 5.5)	DOX, Ce6(Mn)
pH-activated lipid-based nanomaterials	pH-activated lipid-based nanomaterials	Enhanced drug release at low pH	Cancer treatment with axitinib (AXT) and celastrol (CST)	Tumor microenvironment (pH-responsive)	Axitinib (AXT), Celastrol (CST)

#### 5.1.2 Enzymatic activation

Enzymes are pivotal in advancing innovative cancer treatments through the utilization of nanoparticles. These nanostructures are meticulously designed to respond to specific enzymes found in tumor tissues, leveraging the heightened enzyme expression in these areas compared to healthy tissues. Various enzymes, including cathepsins, matrix metalloproteinases (MMPs), glycosyl hydrolases, protein tyrosine kinases (PTK-7), nicotinamide adenine dinucleotide phosphate (NADPH) dehydrogenases (NQO1), and telomerase, have been strategically targeted. For example, cathepsins can break down polymers to release therapeutic agents, while MMPs trigger nanoparticle activation by dissolving protective coatings (22). This enzyme-driven approach significantly enhances the precision of drug delivery, minimizes side effects, and holds great promise for more effective and targeted cancer therapies (Table 4).

**Table 4:** Enzyme-based activation in nanoparticle-mediated cancer treatment (20, 22).

Enzyme	Activation mechanism	Substrate	Findings
Cathepsins	Enzymatic degradation of polymers	Poly-L-lysine hydrobromide (PLL)	PLL-coated gold nanorods and DOX effectively decreased cancer cell viability.
Matrix metalloproteinases (MMP)	Enzymatic degradation of the PVP layer	Gelatin/PVP coating layer	Responsive release of dye molecules in tumors via MMP-9 degradation of PVP coating layer.
Glycosyl hydrolases	Enzymatic degradation of dextrin cap	Dextrin	Dextrin cap degraded by glycosyl hydrolases for controlled drug release in cancerous tissue.
Protein Tyrosine Kinases (PTK-7)	Aptamer interaction and structural change	Aptamer	Aptamer-lipid-PLGA nanoparticles released DOX upon interaction with PTK-7 on tumor cell membranes.
Nicotinamide Adenine Dinucleotide Phosphate (NADPH) Dehydrogenases (NQO1)	Enzymatic decomposition	Prodrug 1	NQO1 decomposes Prodrug 1 into SN-38, a chemotherapy drug.
Telomerase	Telomerase activity-triggered release	Telomerase primer region	Telomerase activity-triggered DOX release decreased cancer cell growth significantly.
Dual enzyme activation	Interaction with MMP-9 and cathepsin B	MMP-9 and cathepsin B	Gemcitabine-poly(ethylene glycol) coated QDs nanosystem for enhanced treatment agent delivery.
Enzyme loaded nanoparticles	Release of enzymes in tumor tissue	Hypoxia-activated prodrug (AQ4N), Glucose oxidase (GOx), Tyrosinases	GOx catalyzes glucose oxidation, enabling AQ4N transformation into a cytotoxic drug. Enzymes are carried into the body via nanoparticles.

#### 5.1.3 Concentration-dependent activation

In the quest for more effective cancer therapies, scientists have harnessed the distinctive attributes of cancer cells to pioneer cutting-edge nanoparticle-based strategies. These ingenious methods capitalize on dissimilarities in gene expression and metabolic processes between healthy and cancerous cells. By zeroing in on membrane proteins such as Transferrin Receptors (TfRs) and Epidermal Growth Factor Receptors (EGFR), researchers have achieved remarkable advancements in tumor penetration and cell apoptosis. Moreover, Prostate-specific membrane antigen (PSMA) has emerged not just as a target but also as a means to enhance nanoparticle uptake. Intriguingly, the oxygen-deprived conditions found within tumors have inspired the development of nano-systems that can trigger therapeutic responses when exposed to low oxygen levels, effectively eradicating cancer cells. Furthermore, fluctuations in intracellular glutathione (GSH) concentrations have enabled the selective release of drugs within cancer cells, while sparing their healthy counterparts (20). These innovative approaches hold tremendous potential in reshaping the landscape of cancer treatment (Table 5).

**Table 5:** Advanced nanoparticles-based cancer-targeting strategies (20).

Target molecule	Nanosystem	Activation mechanism	*In Vitro* results
transferrin receptors (TfRs)	Protoporphyrin IX (PpIX) conjugated human	TfR-mediated endocytic pathway for tumor	Increased penetration of nanosonosensitizers into cancer cells.
			Activation of PpIX by ultrasound, ROS generation.
			Enhanced cell apoptosis in the treatment group.
			Penetration enhancement in multicellular tumor spheroids (MCTS).
Epidermal growth factor receptor	Anti-EGFR antibody conjugated avidin-nucleic-	The binding of ligands leads to receptors and	70% cell death in EGFR over-expressing MDA-MB-231 cells.
			Better cell death compared to the standard ADC with the same DOX concentration.
Prostate-specific membrane antigen	PSMA receptor binding ligand PSMA-1	Accumulation at tumor site through EPR	Almost complete cell kills in PSMA-expressing PC3pip cells upon laser activation.
			35% survival rate in PSMA non-expressing PC3flu cells.
Hypoxia	Multipurpose liposome	Hypoxia-activated theranostic nanosystem	Generation of singlet oxygen in a wider hypoxic tumor environment.
			Activation of AQ4N to its toxic form, killing tumor cells.
Intracellular glutathione (GSH)	Dendritic mesoporous organosilica nanoparticle	Selective release based on GSH concentration	Higher cell kills in cancer cells due to selective release.
			Protection for healthy cells.

### 5.2 Extrinsic activation

Advanced nanotechnology applications are reshaping the landscape of cancer therapy by offering precise targeting, controlled drug release, and innovative treatment modalities. In the domain of Magnetic Hyperthermia, iron oxide nanoparticles (IONs) and gold nanoparticles take center stage (20). When exposed to an alternating magnetic field (AMF), IONs generate heat, leading to enhanced cancer cell death, as evidenced by clinical trials showing significantly improved survival rates compared to conventional treatments (23). Gold nanoparticles, while their heating mechanisms remain the subject of debate, have shown effectiveness in liver cancer cell lines when subjected to AMF. Localized Drug Release techniques harness the power of liposomes, micelles, and novel Fe3O4 core nanoparticles, facilitating controlled drug delivery to tumor sites with exceptional precision and payload efficiency. Smart Stimulus Systems, which include magnetically-driven particles and nanocarriers, introduce the concept of on-demand drug release, potentially revolutionizing therapeutic delivery mechanisms. Light Activation and Photodynamic Therapy (PDT) leverage up conversion nanoparticles, gold nanoclusters, and visible light-activated systems (23). These cutting-edge methods utilize light to initiate localized drug release, generate reactive oxygen species (ROS), and provoke targeted destruction of cancer cells, achieving deep tissue penetration in select cases. x-ray Activation introduces an innovative PDT approach, employing upconverting core nanoparticles to achieve efficient tumor shrinkage with minimal off-target toxicity. Similarly, Titanocene-loaded nano micelles exploit the radiotracer FDG to pinpoint and eliminate cancer cells, resulting in substantial cell death observed in metastatic breast cancer cell lines (24). These diverse nanoparticle-based strategies hold great promise for advancing the field of cancer treatment (Table 6).

**Table 6:** Extrinsic activation of nanoparticles in cancer (20, 23, 24).

Application	Nanoparticle Type	Stimulus	Mechanism	Results
Magnetic hyperthermia	Iron oxide nanoparticles (IONs)	Alternating magnetic field (Amf)	Inductive heating of IONs	Increased cancer cell death; improved survival in clinical trials.
	Gold nanoparticles	Alternating magnetic field (AMF)	Heating through the movement of ions	Promising results in liver cancer cell lines.
Localized drug release	Liposomes and micelles	AMF or permanent magnetic field	Controlled drug release	High specificity and payload efficiency; potential for *in vivo* use.
	Fe3O4 core nanoparticles	Directed magnetic fields	Improved drug delivery and imaging	Increased drug half-life and survival rates in mice models.
Smart stimulus systems	Magnetic particles	External magnetic field	Selective release of drugs	Controlled release mechanism with potential for various therapies.
	Nanocarriers	Heat, NIR, or AMF	On-demand drug release	Targeted and controlled drug release; improved therapeutic effects.
Light activation and PDT	Upconversion nanoparticles (UCN)	NIR or visible light	ROS generation and PDT	Enhanced PDT, deep penetration, and targeted cancer cell destruction.
	Gold nano clusters	NIR	Imaging, gene delivery, and PDT	High uptake and transfection; effective NIR-induced cell death.
x-ray activation	Sr2Al2O4:Eu2+ Core Nanoparticles	x-rays	Efficient tumor shrinkage	Deep penetration and negligible toxicity in vital organs.
	Itanocene-loaded nano micelles	Radiotracer FDG	Targeting GLUT1 protein and PDT	Considerable cell death in metastatic breast cancer cell lines.

## 6 Overcoming drug resistance with nanoparticles

Overcoming drug resistance remains a formidable challenge in the realm of cancer treatment, posing a significant impediment to therapeutic effectiveness and patient outcomes. Nanotechnology has emerged as a promising avenue to combat this issue, leveraging three critical mechanisms: targeting efflux transporters, modulating apoptotic pathways, and addressing the obstacle of hypoxia (25).

### 6.1 Targeting efflux transporters

The notorious efflux transporters, exemplified by P-glycoprotein (P-gp), actively pump drugs out of cancer cells, confounding treatment efforts. Nanoparticles (NPs) play a pivotal role in circumventing these transporters. In stark contrast to free drugs, NPs infiltrate cells via endocytosis, releasing the drug at perinuclear sites, strategically positioned away from the cell membrane and the clutches of efflux pumps. NPs additionally facilitate controlled drug release, often triggered by environmental factors like low pH or redox conditions. Certain NPs, notably polymers, function as multidrug resistance (MDR) modulators, effectively thwarting the action of efflux pumps. By employing combination therapy with NPs as drug carriers, therapeutic outcomes are significantly enhanced, adeptly addressing the challenge of disparate drug pharmacokinetics (26).

### 6.2 Modulating apoptotic pathways

The malfunctioning apoptotic pathways within cancer cells provide them with the means to evade programmed cell death, thereby exacerbating drug resistance. NPs offer a versatile platform for reinstating sensitivity to apoptosis (27). They enable the co-delivery of small interfering RNA (siRNA) targeting anti-apoptotic proteins, such as Bcl-2, in tandem with chemotherapeutics, offering a promising strategy to surmount drug resistance. NP-driven combination therapies can concurrently suppress anti-apoptotic molecules while activating pro-apoptotic compounds, resulting in potentiated therapeutic efficacy. Furthermore, NPs have been adeptly harnessed for the delivery of critical genes like p53, a pivotal tumor suppressor entangled in the intricate web of apoptosis, thereby effectively triggering cancer cell death (28).

### 6.3 Addressing hypoxia

Hypoxia, characterized by reduced oxygen levels within tumors, fuels drug resistance through diverse mechanisms. NPs have emerged as a focal point in research endeavors aimed at mitigating hypoxia-induced resistance. These NPs are engineered to deliver siRNA molecules targeting hypoxia-inducible factor 1α (HIF-1α), a pivotal player in hypoxia-mediated resistance (29). Moreover, inhibitors targeting the HIF-1α signaling pathways, such as PI3 K/Akt/mTOR, can be effectively delivered via NPs, thereby sensitizing multidrug-resistant cells to treatment strategies. NPs further provide an efficacious platform for the delivery of inhibitors targeting heat shock protein 90 (HSP90), which in turn down-regulates HIF-1α expression and successfully confronts resistance associated with hypoxia (30).

## 7 Nanoparticles in clinical translation for cancer therapy

In the age of precision medicine, understanding translational research is crucial for tailoring cancer treatment strategies. Immunotherapy, particularly in the context of nanochemodrugs, has made significant strides, driven by the identification of immune responses against tumor-associated antigens like MUC1, prevalent in breast cancer and adenocarcinomas (31). One promising endeavor is Tecemotide, focusing on MUC1 in Phase III clinical trials for stage IIIA/IIIB NSCLC (Non-small cell Lung Cancer) (32). Lipovaxin-MM, a dendritic-targeted liposomal vaccine, is in Phase 1 trials for malignant melanoma (33). CRLX101, a nanopharmaceutical, employs polymeric nanoparticles (CDP) technology, merging polymeric nanoparticles with recombinant proteins and cholesteryl hydrophobized pullulan (CHP) complexes (34). Trials like IMF-001 targeting the NY-ESO-1 antigen show promise. Combining this vaccine with PD-1 (Programmed Cell Death Protein 1) blockade exhibits potential in human trials (35). Nanochemodrugs, notably Nab-paclitaxel, with high drug-binding capacity due to nanoparticle-conjugated albumin (Nab), are being tested in conjunction with gemcitabine, atezolizumab, and cyclophosphamide for metastatic and early-stage breast cancer (36). ABI-007, combining Nab-paclitaxel, is undergoing clinical trials for stage IV NSCLC and metastatic breast cancer (37). Table 7 summarizes nanoparticle formulations undergoing clinical evaluation across various cancer types, exemplifying the potential of nanochemodrugs in advancing cancer treatment.

**Table 7:** Nanodrugs that have undergone clinical trials (47).

Nanodrug	Conventional drug	Cancer type	Clinical trial identifier
Cyclodextrin-based polymer	Camptothecin	NSCLC primary peritoneal cancer	NCT01380769
CPC634 (CriPec®)	Docetaxel	Ovarian cancer	NCT03742713
Nab-rapamycin (ABI-009)	Rapamycin	PEComa	NCT02494570
		Non-muscle-invasive bladder cancer	NCT02009332
		Solid tumours	NCT00635284
Aldoxorubicin (DOXO-EMCH, INNO-206)	Doxorubicin	Advanced solid tumour	NCT01673438
		Glioblastoma	NCT02014844
		Pancreatic ductal adenocarcinoma	NCT01580397
Cavrotolimod (AST-008)	AST-008 (toll-like receptor 9 agonist oligonucleotide)	Healthy volunteer study	NCT03086278
	AST-008, Pembrolizumab, Cemiplimab	Solid tumours; melanoma; head and neck squamous cell carcinoma; cutaneous squamous cell carcinoma; Merkel cell carcinoma	NCT03684785
Cyclodextrin–PEG copolymer nanoparticle	Camptothecin (Topoisomerase I inhibitor)	Metastatic castration-resistant prostate cancer	NCT02010567
			NCT03531827
AGuIX	Polysiloxane gadolinium-chelates based nanoparticles	Brain metastases	NCT02820454
Docetaxel-PNP	Taxotere	Solid tumors	NCT02274610
Paclitaxel Nab	Carboplatin, Erlotinib hydrochloride	NSCLC	NCT00661193
	Bevacizumab, Gemcitabine hydrochloride	Breast cancer	NCT00623233
	Carboplatin, Herceptin®	Breast cancer	NCT00093145
	Cetuximab, Cisplatin	Cetuximab, Cisplatin	NCT00833261

Nanomaterials have the potential to transform cancer treatment by allowing for targeted drug delivery to cancer cells, thereby minimizing harm to healthy tissues. They can be modified with ligands that attach to specific receptors that are often overexpressed on cancer cells, which helps reduce chemotherapy side effects and enhances drug effectiveness. Various nanocarriers, including liposomes, micelles, and polymeric nanoparticles, can release their therapeutic contents in response to specific triggers such as pH, temperature, or enzymes found in the tumor microenvironment, thereby increasing precision. Additionally, nanomaterials can be employed to deliver cancer vaccines that activate the immune system to recognize and attack cancer cells. Nanoparticles can improve the presentation of antigens to immune cells, thereby enhancing the body's natural immune response (38).

Nanoparticles can be created from various materials, including metals, semiconductors, and polymers. The processes for producing nanoparticles are generally categorized into two main approaches: top-down and bottom-up. Top-down methods involve reducing larger materials into nanoparticles through physical and chemical techniques. For instance, mechanical milling utilizes mechanical energy to break down larger particles into nanoscale sizes, with high-energy ball milling being a common technique. Lithography is employed to carve nanoscale patterns from larger structures, often using light, ion beams, or electron beams. Another technique, laser ablation, focuses a powerful laser beam on a solid material, vaporizing it to create nanoparticles. Conversely, bottom-up approaches involve constructing nanoparticles atom by atom or molecule by molecule. In chemical vapor deposition, gases are broken down on a heated surface to produce thin films or nanoparticles. The sol-gel method involves a liquid precursor that undergoes hydrolysis and condensation reactions, forming solid nanoparticles in a liquid medium. Colloidal synthesis involves reducing metal salts in a solution, which is a prevalent method for generating gold and silver nanoparticles. Furthermore, nanoparticles can be designed for targeted drug delivery, enhancing treatment efficacy while minimizing side effects (39, 40).

## 8 Future prospects

Nanoparticles have become a focal point in the field of medicine, offering promising prospects for drug delivery and diagnostics. However, the potential long-term health effects stemming from prolonged nanoparticle exposure have raised growing concerns among researchers. These concerns are rooted in the distinctive characteristics of nanoparticles, particularly their size and ability to penetrate biological systems (2) To gain a comprehensive understanding of the developmental and neurobehavioral impacts of medical nanoparticles, there is a pressing need for thorough research. Unfortunately, the relatively recent emergence of nanoparticles in medicine has impeded the establishment of effective regulatory methods to evaluate their potential risks (41).

In cancer diagnosis and therapy, nanotechnology has ushered in transformative changes by augmenting drug delivery and enabling precise targeting within the body. For example, scientists can craft passive or active nanostructures to deliver drugs to remote anatomical locations that were previously inaccessible to conventional macromolecular medications (42). An example of this innovative approach is the utilization of the nanoFOD (fiber-optic dosimeter) device, which leverages nanomaterials to accurately locate and measure radiation doses in real time during external beam radiation therapy sessions (43). The prospects presented by nanorobotics and molecular nanosystems are equally captivating, envisioning the creation of artificial organs and system mimics, potentially revolutionizing the landscape of nanochemotherapy (44). Researchers are actively exploring the “*safe-by-design*” concept for nanomaterials, which holds significant importance for pharmaceutical companies. This concept revolves around cost-effective risk management, achieved by integrating safety and risk assessments into the early stages of product development (45).

## 9 Challenges encountered

Nonetheless, as nanotechnology continues to advance in medicine, several limitations and challenges come to the forefront. One primary concern revolves around the potential long-term health repercussions of nanoparticles. Researchers are increasingly alarmed about the possible adverse effects arising from extended exposure to these tiny particles, given their unique attributes, including size and penetration capabilities (46). Consequently, in-depth investigations into the developmental and neurobehavioral consequences of nanoparticle use in medicine are imperative. While precision medicine offers the tantalizing prospect of tailoring treatments to individual patient profiles based on their disease susceptibility and treatment responses, it is not without its complexities. For example, the combination of antioxidants with chemotherapy, although promising for cancer prevention, has spurred debates within the medical community due to potential unintended consequences. Rigorous research is essential to identify the optimal combinations and dosages of antioxidants for various cancer types, ensuring both safety and efficacy (47).

In the domain of nanotechnology-based cancer diagnosis and therapy, significant strides have been made, yet numerous hurdles remain. A crucial challenge lies in ensuring the reliability of nanotechnology-based diagnostic tools when applied in clinical settings. Consistency and accuracy are paramount, necessitating the resolution of issues like nonspecific nanoparticle probe binding, aggregation, and inappropriate detection methods through extensive clinical trials and ongoing efforts Another obstacle is the cost-effective large-scale production of sensitive nanoprobes. Although many nanoprobes are developed in meticulously controlled laboratory settings, achieving batch consistency and cost reduction remains a significant challenge (48). Streamlining synthesis procedures and enhancing nanoprobe functionalization are essential steps in achieving batch uniformity and cost reduction. Additionally, evaluating the cost-effectiveness of developing nanotechnology-based platforms is crucial, as not all nanotechnology-based tests developed in academic laboratories are suitable for clinical use. These challenges underscore the need for practical and cost-effective solutions (49).

The clinical application of nanoparticles encounters various regulatory challenges that can hinder or complicate their approval for medical use. These challenges involve characterization, standardization, safety, toxicology, market access, and post-market surveillance. Overcoming these obstacles necessitates collaboration among researchers, industry players, and regulatory bodies to create clear guidelines and protocols that promote the safe and effective clinical application of nanoparticles (50).

Nanoparticles pose potential risks to human health and the environment due to their small size, high reactivity, and long-lasting presence. They can contaminate ecosystems, water, and soil. Large-scale production is challenging, expensive, and time-consuming, and nanoparticles tend to clump together, reducing their effectiveness. There's limited knowledge about their long-term effects. However, these issues can be addressed before marketing approval by developing nanostructures with the right properties, ensuring reproducible manufacturing, using appropriate analysis methods, and demonstrating safety, efficacy, and a favourable toxicity profile through clinical trials (51–55).

## 10 Conclusion

The integration of nanotechnology into cancer diagnosis and therapy stands as a transformative frontier in oncology. Nanoparticle-based drug delivery systems have demonstrated remarkable potential in elevating treatment effectiveness while simultaneously reducing systemic toxicity, providing renewed hope for individuals battling cancer. These pioneering approaches span a wide spectrum of applications, encompassing chemotherapy, targeted therapy, radiotherapy, hyperthermia, and gene therapy. The adaptability of nanomaterials enables the creation of hybrid platforms with augmented properties, offering the promise of more precise and efficient cancer treatment. Nonetheless, it remains imperative to confront challenges, including potential toxicity, resource-intensive processes, and the need for robust reliability, to facilitate the clinical translation of these innovations. Ongoing research endeavors should prioritize the refinement of nanoplatforms, aiming not only to target cancer cells but also to engage with the tumor microenvironment and the immune system. This approach holds the potential to foster personalized and highly efficient therapeutic strategies. By harnessing the unique attributes of nanomaterials and continuously enhancing their safety and efficacy profiles, we can envision a future where nanomaterial-based therapies assume a pivotal role in revolutionizing cancer management, ultimately benefiting countless cancer patients across the globe.
